# Allosteric Modulation of Neurotransmitter Transporters as a Therapeutic Strategy

**DOI:** 10.1016/j.tips.2020.04.006

**Published:** 2020-05-26

**Authors:** Marco Niello, Ralph Gradisch, Claus Juul Loland, Thomas Stockner, Harald H. Sitte

**Affiliations:** 1 Centre for Physiology and Pharmacology, Institute of Pharmacology, Medical University of Vienna, Vienna, Austria; 2 Laboratory for Membrane Protein Dynamics. Department of Neuroscience. University of Copenhagen, Copenhagen, Denmark; 3 AddRess, Centre for Addiction Research and Science, Medical University of Vienna, Vienna, Austria

## Abstract

Neurotransmitter transporters (NTTs) are involved in the fine-tuning of brain neurotransmitter homeostasis. As such, they are implicated in a plethora of complex behaviors, including reward, movement, and cognition. During recent decades, compounds that modulate NTT functions have been developed. Some of them are in clinical use for the management of different neuropsychiatric conditions. The majority of these compounds have been found to selectively interact with the orthosteric site of NTTs. Recently, diverse allosteric sites have been described in a number of NTTs, modulating their function. A more complex NTT pharmacology may be useful in the development of novel therapeutics. Here, we summarize current knowledge on such modulatory allosteric sites, with specific focus on their pharmacological and therapeutic potential.

## Neurotransmitter Transporters – Key Regulators of Synaptic Transmission

**Figure 1:**
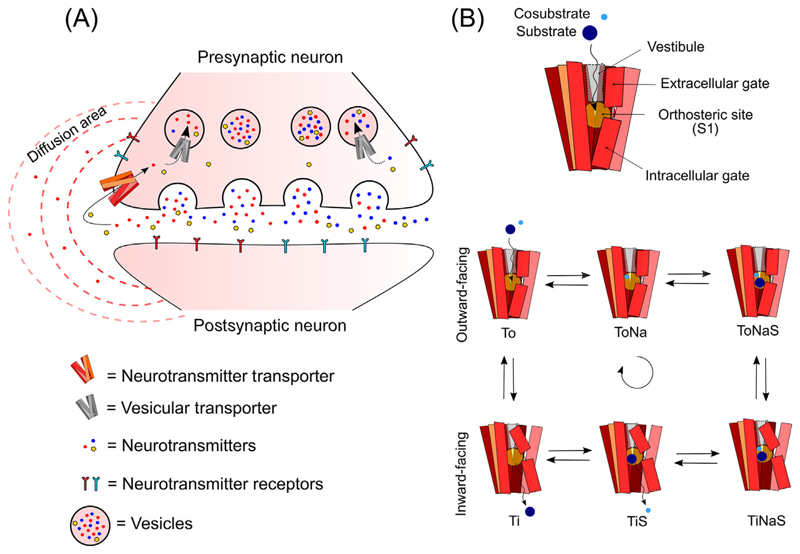
Neurotransmitter Transporters (NTTs) Regulate the Neurotransmitter Signal. (A) Neurotransmitters are rapidly removed from the synapse by NTTs present in the presynaptic neurons (red transporter), after being released into the extracellular space. By exerting their functions, NTTs regulate the concentration of neurotransmitters available for pre- and postsynaptic receptors. Depending on the activity of the NTT, the diffusion area of the neurotransmitter can change and influence cellular processes far from the releasing site. Once reuptake into the presynaptic neuron has occurred, the neurotransmitter is rapidly removed from the cytoplasm and packed into vesicles by vesicular transporters (grey transporter). (B) NTTs have a common general structure composed of a vestibule, leading to the orthosteric site. The orthosteric site is located between an extracellular and intracellular gate (top panel). Before binding the substrate and the cosubstrate, the NTT is in its outward-facing conformation (‘To’, bottom panel). In this conformation the transporter binds first Na^+^ (‘ToNa’) and then the substrate (‘ToNaS’). Together, these binding events allow the NTT to flip from the outwardfacing conformation to the inward-facing conformation (‘TiNaS’). Na^+^ and S can now be released in the cytoplasm (‘TiS’ and ‘Ti’) and the NTT can return to the outward-facing conformation.

Synaptic transmission is a fundamental feature of the communication between neurons [1]. After the quantal presynaptic release of neurotransmitter into the synaptic cleft, binding to pre- and postsynaptic receptors follows. Subsequently, the signal is quickly terminated by efficient reuptake of the neurotransmitter back into the presynaptic neuron (Figure 1A). Reuptake is tightly regulated by neurotransmitter transporters (NTTs), a class of complex molecules, each holding specificity for only one or a few neurotransmitters. For instance, the dopamine and norepinephrine transporter (DAT and NET, respectively) can efficiently uptake both dopamine and norepinephrine with similar kinetics [2,3], while the serotonin transporter (SERT) can efficiently transport serotonin but only binds dopamine with very low affinity [4]. Once inside the presynaptic neuron, the neurotransmitters are, in general, rapidly confined into vesicles by specific vesicular transporters (Figure 1A, grey transporter), in order to prevent toxicity [5]. The rapid removal of neurotransmitters from extracellular fluids avoids an exaggerated signal and confines the neurotransmitters within the intended area, preventing chemical crosstalk [6] (Figure 1). Because of their important role in synaptic transmission, dysfunctional or improper regulation of the NTTs contribute to neurological and neuropsychiatric disorders [7]. Therefore, NTTs are also clinically relevant pharmacological targets for the treatment of major neuropsychiatric diseases, such as depression, attention-deficit hyperactivity disorder, epilepsy, and narcolepsy [8].

### General Structure and Mechanism of Function of NTTs

All NTTs require the concentrating power of specific ions in order for the substrate to accumulate on its respective side of the membranes. The type and the stoichiometry of different ions vary among NTTs. In general, Na^+^ and Cl^–^ are required in the case of DAT, NET, and SERT, and Na^+^ and K^+^ in the case of the excitatory amino acid transporters (EAATs) 1–5. Ionic concentration underlies the ability of the NTTs to accumulate substrate on one side of the cellular membrane [8,9].

NTT structure can vary quite a lot between different classes of transporter, for instance, DAT, NET, and SERT are organized in 12 transmembrane helixes (TM), while other NTTs such as EAATs are instead organized in eight TMs. These TMs are connected by extracellular and intracellular loops, which work as regulatory sites through different post-translational modifications such as phosphorylation and glycosylation [10,11]. As far as NTTs are concerned, the 3D organization of the different TMs and loops determine a complex structure composed of a vestibule leading to the central binding site confined between an extracellular gate and an intracellular gate (Figure 1B) [8,12]. The dynamics of this structure follow the so-called ‘alternate access model’ upon binding of substrate and ions, an extracellular gate seals off the binding site and the protein alternates conformation between outward- and inward-facing modes, resulting in the transport of the substrate across the membrane [13] Figure 1B, bottom). This alternating process has been suggested to pass through different intermediate configurations, which are regulated in concert by the substrate and ions and is known as ‘the transport cycle’ [14,15].

### Transport Cycle and Transporter-Mediated Currents

NTTs are secondary active transporters and therefore they couple substrate transport to the electrochemical gradient of Na^+^. Most of them cotransport Cl^–^ and some of them counter-transport K^+^ or H^+^ [8].

In the simplified version of the transport cycle (see Figure IC in Box 1), the transporter in the outward-facing conformation (To) needs to first bind Na^+^ (ToNa) and then the substrate (ToNaS) before it can transition to the inward-facing conformation (TiNaS). At this point, since the internal concentration of Na^+^ and S is very low, they can dissociate from the transporter (TiS and Ti) and the transporter in the inward-facing conformation can now return to the outward-facing conformation (To). In some cases, other ions (e.g., K^+^ and/or H^+^ in the case of SERT [16]) can modulate the dissociation of the substrate (S) internally and therefore regulate the efficiency of the transport process.

The coupling between substrate and ions during the transport process results in the so-called ‘transporter-mediated currents’ that can be monitored by means of a whole-cell patch clamp (for a detailed review, consider [17]).

The typical transporter-mediated current is an inwardly-directed current. These are voltagedependent and can be elicited by applying the substrate with a perfusion device able to switch between different solutions within 50–100 ms [16,18,19]. When cells are clamped at negative potential and under physiological conditions, they show a so-called ‘steady-state current’, which is present until the substrate is continuously applied to the cell, then decays back to the baseline as soon as the substrate is no longer present (see Figure IC in Box 1). This has been shown to be representative of the transporter completing the transport cycle and is therefore an average of all the charge movements happening during the transport cycle (see Figure IC in Box 1, bottom, blue and red). In some transporters, a peak current can also be present (see FigureIC in Box 1, top, red). If present, the peak current contains information about the outward-open to the inward-open transition and it can be elicited either by substrate application or by binding of inhibitors [20–23].

The whole-cell patch clamp gives access to both the intracellular and the extracellular component. By substituting different ions or introducing compounds into the patch pipette, it is possible to isolate different states of the transport cycle and extract rate constants of all these partial reactions. These constants can then be used for developing kinetic models of the transport cycle [15]. Detailed kinetic models exist already for GABA transporter-1 (GAT-1) [24], DAT [25], SERT [16,26,27], and glycine transporters GlyT1 [28] and GlyT2 [28]. Keeping in mind the information present in such kinetic models during drug development may help in understanding differences between apparently similar drugs and transporters and provide information about allosteric effects [14,19,29,30].

### NTTs: The Input/Output Neurotransmission System

Not only do transporters participate in the reuptake of neurotransmitters, shaping the input signaling (i.e., receptor activity), but they can also reverse their transport direction and release neurotransmitters in an exocytosis-independent manner and therefore participate in output signals. This process, also known as transporter-mediated ‘efflux’, has been mainly described for pharmacological agents like amphetamine and congeners targeting monoamine transporters [31]. As substrates of these transporters, they interact with the proton-driven vesicular monoamine transporters (VMATs) and are translocated into vesicles, dissipating their proton gradient and redistributing the vesicle content [32] (Figure 1A). Concomitantly, amphetamines inhibit the activity of monoamine oxidases (MAO) [33], catechol-O-methyl-transferase (COMT), and activate different kinases (e.g., alpha-CamKII or protein kinase C [34]). The combined actions on the VMAT, MAO, and COMT determine the cytosolic increase in the endogenous neurotransmitter (e.g., dopamine). When the concentration of neurotransmitter is high enough in the presence of Na^+^, it can bind to the intracellular side of the NTT and translocate to the extracellular space [31].

In the case of SERT, this pharmacologically induced efflux has been recently suggested as a required mechanism for the prosocial effect of 3,4-methylenedioxy-N-methylamphetamine (MDMA), also known as ‘ecstasy’ [35], which is currently under clinical evaluation for the treatment of social anxiety symptoms in post-traumatic disorder [36] and autism [37].

However, efflux can also happen in a pharmacologically independent way. During ischemia, ATP levels decrease and affect the function of the Na^+^/K^+^-ATPase. This leads to an increase of extracellular K^+^, which in turn reverses glutamate transport and thereby triggers neuronal death due to excitotoxicity [38]. In the case of early-onset Parkinsonism, a coding variant has been identified, the DAT-Asp421Asn, characterized by a constitutive, anomalous dopamine efflux [39]. A similar alteration in the coding variant DAT-T356M has also been identified in autism [40,41].

Taken together, these studies suggest that NTT-mediated efflux could be potentially targeted for the development of future medications. However, future research will be required to expand on this important function of NTTs.

### The Complex Pharmacology of NTTs

The complexity of the NTT–ligand interaction can be gauged by several examples: amphetamine, for instance, is a substrate of monoamine transporters [31]; however, amphetamine is often described as an inhibitor because of its inhibitory action on dopamine transport. Amphetamine binds to the orthosteric binding site of DAT and NET, thus acting as a competitive inhibitor by preventing dopamine and norepinephrine from being transported [31]. Cocaine is a nontransported psychostimulant and stabilizes DAT, NET, and SERT in an outward-open conformation by binding to the orthosteric site [42], an example of a classical competitive inhibitor that also prevents uptake of physiological substrates. Ibogaine, however, is another nontransported psychostimulant that was initially recognized as an inhibitor binding preferentially to an inward-facing conformation of SERT [26,43] and suspected to bind to an alternative binding site [44]. The recent structure of SERT in complex with ibogaine, however, clearly shows an ibogaine molecule bound to the orthosteric site [45]. Hence, it has now to be classified as an atypical, noncompetitive inhibitor [46]: it induces a conformational transition to the inward-facing state (by contrast to cocaine [44] and other competitive inhibitors like paroxetine [26]). The orthosteric binding site is thereby stabilized in an inward-facing conformation, which is not accessible from the extracellular side, thus leading to this complex behavior of noncompetitive inhibition [45].

Hence, there is considerable heterogeneity among the ligands that bind to NTTs; the wide range of these obviously orthogonally differing interaction patterns results in a rich pharmacology [46]. Moreover, given the pharmacological importance of this protein class, there is potential to develop agents with a different pharmacological function, such as **use-dependency** (see Glossary) or enhancement of transport activity: only allosterically bound compounds would be able to provide such promising features.

## The Principles of Allosteric Modulation

**Figure 2:**
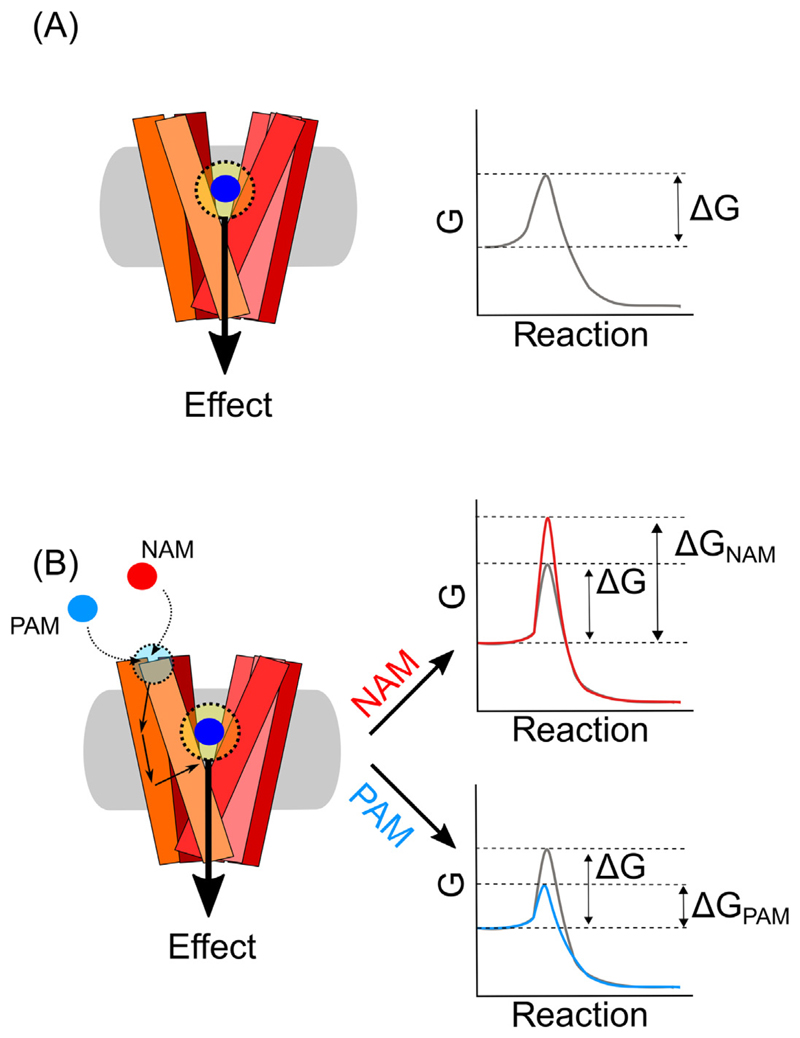
Allosteric Modulators and the ‘Action-at-a-Distance’ Structural and energetic view. (A) Comparison between the effect mediated by a ligand (blue) interacting with the orthosteric site (yellow, left) and the ‘action-at-a-distance’ mediated by a ligand (cyan, right) interacting with an allosteric site (light cyan, right). The ‘action-at-a-distance’ can be seen from a structural or a nonstructural point of view. From a structural point of view, the binding of the ligand determines a cascade of conformational changes that can be sensed by the different domains and transmitted to the orthosteric site (unbroken arrows). From a nonstructural point of view instead, allostery may determine its effect by an infinite number of microstates that include changes in entropy and enthalpy, represented by changes in the Gibbs free energy (ΔG). (B) In order to elicit an effect, the enzymatic reaction has to overcome its own energetic barrier (ΔG, grey). Allosteric ligands increase or decrease the ΔG of the reaction according to their activity as negative- or positive allosteric modulators (NAM, red or PAM, blue) respectively.

Allosteric modulation or allostery is a widespread phenomenon; one of the first reports was the feedback regulation in deaminases [47,48]. In general, ligands bind to a protein within an inherent binding site, the orthosteric site. However, proteins may also possess one or more alternative binding sites not targeted by the endogenous or common compounds. These are termed secondary or allosteric sites [49,50]. Ligand binding to an allosteric site transmits an effect (positive or negative) to other parts of the protein, thereby modulating function and/or activity of the orthosteric site, leading to an ‘action-at-a-distance’ (Figure 2A). Accordingly, allosteric ligands likely change the conformation or the conformational dynamics of the protein and thereby modulate the access or binding of ligands to the orthosteric site. However, other activity-modulating possibilities have also been proposed, such as that allosteric binding leads to a change in the energetic barrier (represented by ΔG), thus affecting the kinetics of biological processes [51] Figure 2B). In some instances, allostery cannot be easily explained, as seen with the example of the complex behavior of ibogaine (earlier). Box 2 provides a brief overview of allostery models proposed in the field. In transport proteins, the situation is more complex as ligands not only bind to proteins, but they may be translocated to the intracellular side by inducing the transporter transition from an outward-facing to an inward-facing conformation. Thus, allosteric ligands may increase the intrinsic transport rate, induce or inhibit reverse transport operation, termed efflux, or simply bind to block or slow down protein function.

The use of allosteric modulators rather than orthosteric ligands offers several pharmacological and therapeutic advantages:

(i) Ligands for the orthosteric site are often analogs of the endogenous ligand and accordingly cause potential side effects by also affecting other proteins targeted by the same endogenous ligand. Allosteric binding sites do not possess the same structural conservation and could have higher specificity with fewer side effects.
(ii) Allosteric modulators do not have to be activators per se, but could change the activity of the protein’s intrinsic function in specific conditions (e.g., in the presence of the endogenous ligand).
(iii) They can also possess use-dependency, being more active modulators when the proteins have increased activity [52].

### Approaching the Energetic Dimension of Allostery

Several approaches have been developed to investigate the allosteric nature of ligands. Simple analytical approaches such as the Hill-Langmuir equation can be used to obtain an indication of allostery [53]. Different methods are described in Box 1. Observations based on these methods have led to the development of general models for describing the binding of ligands to proteins and allostery.

The first model proposed for allostery was the ‘conformational selection model’. This model assumes that the high affinity relaxed (R) state and the low affinity tensed (T) state of a protein, pre-exist in equilibrium for a protein homo-oligomer with tightly coupled protomers [54]. Although other models have been developed, the conformational selection model is the one that obtained the largest support and was subsequently expanded (Box 2). The development of new computational approaches opened the way to a more in-depth study of allosteric connectivity or pathways from an energetic point of view: if a state within one domain undergoes conformational changes, the interaction energy can either decrease or increase and therefore stabilize or destabilize a conformation in a second domain [55]. The differences in energy between states can be mathematically described to understand the free energy contribution for the transition between different possible states. Accordingly, the change in interaction energy allows individual domains to sense each other [55]: when a ligand binds to the high affinity state, the free energy of ligand binding stabilizes this state and leads to a redistribution of forces and conformations within the protein ensemble. Binding of an effector molecule stabilizes the effector binding domain, which can in turn stabilize (positive coupling) or destabilize (negative coupling) the high affinity state in the second domain [55]. Entropy and changes in entropy should therefore also be included, as allostery can originate from changes in protein dynamics or conformational distribution upon ligand binding, without large changes in enthalpy [56].

The field of G protein-coupled receptors (GPCRs) has been at the forefront in development and discussion of theoretical aspects of allostery in membrane proteins [50,57–59]. It has not received much attention in the case of NTTs. However, many of the basic allosteric principles in GPCRs do not apply to transport proteins, hence some new concepts have to be introduced.

### Allosteric Modulators of NTTs: Making Sense of the Current Knowledge

Recent studies on the pharmacology of NTTs have established the heterogeneity among compounds as described earlier, which initially called to mind the GPCR pharmacology complexity [11,46,60]. In the case of NTTs, only a few crystal structures are available and only a small part of them show compounds bound in allosteric sites [61,62]. In addition, only in a few cases has the position of the compounds been confirmed by various complementary methods, such as photoaffinity labeling, mutagenesis studies, or binding experiments using radiolabeled ligands. Indeed, all these methods, including crystallization, need to be considered carefully and supported by individual accessory methodologies. To date, there are only a few examples where information on the exact binding locus in allosteric sites exists (see later). Therefore, this needs to be declared as one of the most important outstanding questions.

### The Structural Heterogeneity of Allosteric Modulation in NTTs

**Figure 3:**
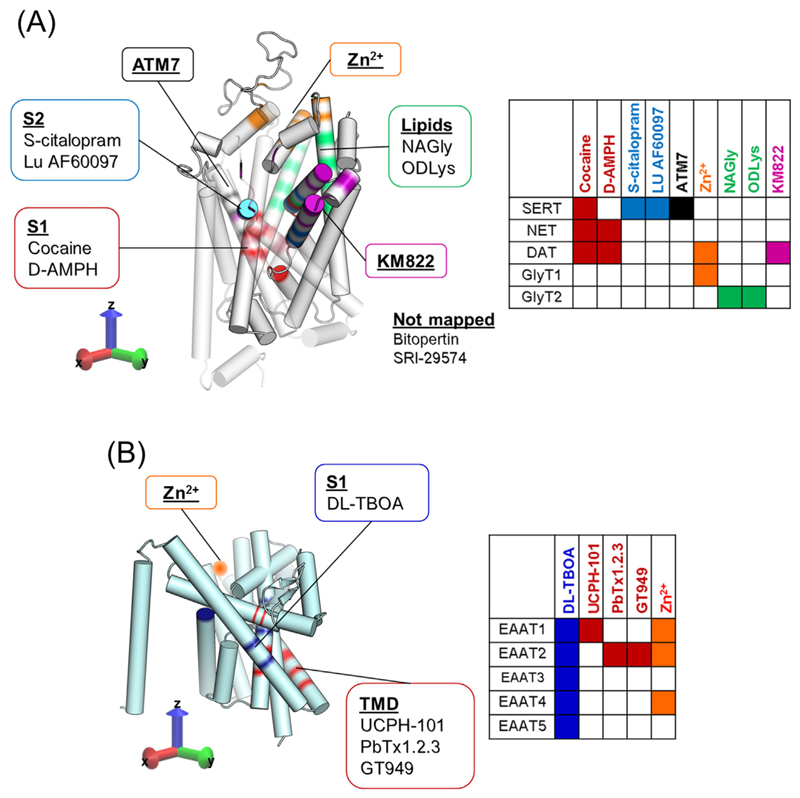
Key Figure Localization of Orthosteric and Allosteric Binding Sites in the Two Main Classes of Neurotransmitter Transporters (NTTs) (A) Structure of solute carrier 6 (SLC6) transporter family, which includes the transporters for serotonin, norepinephrine, dopamine, and glycine; orthosteric and allosteric binding sites that have been described to date are highlighted in different colors. Examples of compounds modulating such sites are also shown. Red: orthosteric binding site, also referred to as S1-site, with two examples of compounds binding these [e.g., cocaine and D-amphetamine (D-AMPH) in monoamine transporters (MAT)]. Cyan: S2-site in serotonin transporter (SERT) has been shown to be targeted by S-citalopram and Lu AF60097. Black: additional binding site recently proposed in SERT with its allosteric modulator ATM7. Orange: Zn^2+^ binding site. Green: allosteric binding site in GlyT2 amenable to targeting of specific acylamino acids, such as NAGly and ODLys. Purple: binding site described in the dopamine transporter (DAT) and modulated by KM822. In the middle are highlighted the compounds shown to act as allosteric modulators, but whose structural determinants are still uncharacterized. The table at the bottom highlights which transporter is targeted by the respective inhibitor in the tridimensional map of SLC6 transporters (color code matched). (B) Structure of solute carrier 1 (SLC1) transporter family, including various excitatory amino acid transporters (EAATs 1-5); orthosteric and allosteric binding sites that have been described to date are highlighted in different colors. Blue: central binding site, targeted, for instance, by the nonselective and competitive inhibitor of the EAATs, DL-threo-β-benzyloxyaspartic acid (TBOA). Red: trimerization domain (TMD) shown to bind UCPH-101 in EAAT1 and suggested as binding site for PbTx1.2.3and GT949in EAAT2. Orange: Zn^2+^ binding site proposed for EAAT1, EAAT2, and EAAT4. The table at the bottom highlights which transporter is targeted by the respective inhibitor in the tridimensional map of SLC1 transporters (color code matched).

Drugs acting at NTTs may be divided into those binding to the orthosteric or S1-site, the vestibule of the transporter, the S2-site, or to sites outside of the transport pathway (e.g., Zn^2+^-binding in DAT and in EAATs) or to the oligomerization domain (e.g., UCPH-101 in EAATs). Ligands and their respective binding site are shown in Figure 3 (Key Figure).

If, on the one hand, this structural classification helps to compare the pharmacology of different NTTs, it does not provide any information from a functional point of view. In the case of GPCRs, it has been established that compounds may either be classified as negative or positive allosteric modulators (NAMs or PAMs) [50]. While this terminology may still be used in the case of NTTs, the coexistence of separate functional processes (i.e., influx and efflux) implies that a compound does not necessarily affect both processes in the same way. A compound working as a NAM on uptake may have no effect, a similar effect, or an opposite effect when it comes to efflux. Also, a compound that is a NAM on substrate transport may enhance the binding of an orthosteric bound inhibitor and, therefore, act as a PAM in that respect (Box 3). For the sake of simplicity, in the following we will only classify NAMs and PAMs of uptake; since there are only a few modulators of efflux, they are briefly described in Box 3.

#### NAMs: Simply Inhibiting Uptake?

The existence of allosteric sites in SERT was postulated almost four decades ago [63,64], based on the observation that the dissociation of a prebound radiolabeled tracer was inhibited. The rate of ligand dissociation is a function of the intrinsic stability of the protein–ligand complex. If a second ligand reduces the dissociation rate of the first ligand, then this is most likely caused by an allosteric mechanism, in which the second ligand increases the energetic barrier of ligand dissociation by a conformational change, by further stabilizing the bound state or just obstructing the path of ligand dissociation. In SERT, a variety of ligands were described to inhibit the dissociation of [^3^H]citalopram. The most potent one was the selective serotonin reuptake inhibitor citalopram, but also the related compounds paroxetine and sertraline showed significant effects, while fluoxetine, venlafaxine, and duloxetine had no effects [65,66]. Serotonin has also been shown to exert an allosteric effect on the dissociation of [^3^H]imipramine, although with a very low potency [67]. Even though these early observations supported the existence of two binding sites in SERT, it was not until three decades later that the sites were mapped: first, the orthosteric or S1-site in the center of the protein [68] and subsequently, the allosteric or S2-site in the extracellular vestibule, located in the entry path to the orthosteric binding site S1 [66] (Figure 3A). The allosteric effect was proposed to be due to steric hindrance of the exit pathway for the S1-bound radioligand. The molecular models were substantiated by mutagenesis of residues in the S2-site, decreasing allosteric binding. Indeed, SERT-G402H completely ablated allosteric binding of the tested ligands [66]. Atomic force microscopy (AFM; Box 1) experiments using S-citalopram as the ‘probe’ [69] and the crystal structure of human SERT [61] confirmed the presence of two citalopram binding sites in SERT. However, by contrast to previous [^3^H]citalopram dissociation experiments [65], no sertraline moieties have been found in the S2-site [70].

To date, all currently known selective serotonin reuptake inhibitors and tricyclic antidepressants such as clomipramine bind to the S1-site with higher affinity than to the S2-site [64]. The most potent S2-bound ligand was S-citalopram, but its affinity is still about 5 μM, approximately 1000-fold lower than its affinity for the S1-site (Kd ~5 nM). Recently, a ligand (Lu AF60097) was found that had an S2-site affinity of ~30 nM and an S1-site affinity of around 300 nM [71] (Figure 3A). These results show that it is possible to generate compounds with both high S2-site affinity and selectivity relative to the S1-site, opening the possibility of approaching therapeutic effects by targeting the S2-site. Moreover, the study shows that Lu AF60097 was able to potentiate the binding of imipramine to SERT, opening the possibility of decreasing its dosage and thus alleviating some of the detrimental side effects of imipramine treatment [71].

A similar site has also been hypothesized for DAT and NET. However, none of the tested compounds could slow down the dissociation of the prebound GBR12935, which binds to the orthosteric site in DAT, and only sertraline could slow down the dissociation of [^3^H]nisoxetine in NET [72]. Recently, it was reported that two quinolone analogs, SoRI-9804 and SoRI-20040, are able to inhibit both uptake and efflux of [^3^H]-dopamine, but only with partial efficacy (40–60%). The partial efficacy is present even at saturating concentrations, suggesting that its binding must halt the catalytic efficiency [i.e., maximal transport velocity (*V_max_*)], without being a competitive inhibitor [73]. Similar to allosteric activity of S-citalopram in SERT, SoRI-9804 and SoRI-20040 reduced the dissociation of a radioligand prebound in the orthosteric site of DAT [74]. By contrast, SoRI-20041 partially inhibits dopamine uptake without affecting DAT-mediated release [73]. Subsequently, the SoRI-20041 structure was optimized in order to increase the affinity for DAT. This resulted in SRI-29574, a high-affinity DAT inhibitor, showing similar pharmacological features to its parent compound [60]. The binding site of these compounds is still unknown, but regardless of their potential clinical application, their unconventional transporter pharmacology may allow better elucidation of the complex transport mechanism of NTTs.

By using an in silico-based approach, the ligand KM822 was recently identified as an allosteric inhibitor of DAT (Figure 3A) [75]. KM822 shows a fairly good selectivity towards DAT and stabilizes it in the outward-open conformation. Saturation of [^3^H]-dopamine uptake experiments show a mixed profile as voltammetry experiments performed in acute striatal slices demonstrate that KM822 reduces cocaine inhibition of dopamine uptake without affecting dopamine release. Moreover, KM822 reduces the psychostimulant-mediated increase in locomotion, suggesting it as a possible candidate to treat addiction [75].

By contrast, the GlyT inhibitor bitopertin is promising from a clinical point of view [76]. GlyT1 regulates glycine dynamics at both inhibitory and excitatory synapses. Indeed, glycine acts on excitatory synapses as a coagonist with glutamate at N-methyl-d-aspartic acid (NMDA) receptors and on inhibitory synapses, activating the inhibitory glycine receptors [77]. Bitopertin shows selectivity towards GlyT1 over GlyT2 and reduces V_max_ of [^3^H]glycine uptake without changing its K_m_, an indication of allosteric inhibition [78,79]. Bitopertin normalized amphetamine-induced hyperlocomotion and phencyclidine-induced amphetamine-sensitization, indicating its ability to modulate a state of dopaminergic dysregulation [79]. This effect has been linked to the ability of glycine to potentiate NMDA-stimulated GABA release in the corpus striatum, which in turn would inhibit dopamine release [80]. However, despite these promising early results, bitopertin did not pass Phase III clinical trials for the treatment of negative symptoms of schizophrenia [81,82]. Nevertheless, the outstanding preclinical and early clinical data of bitopertin led to its investigation in combination with other drugs^i^ and for the treatment of other conditions^ii^.

Furthermore, promising results for the treatment of chronic pain [83] have been obtained by noncompetitive inhibition of GlyT2 by N-arachidonyl-glycine (NAGly) at low μM affinity [84]. More recently, analogs of NAGly with nanomolar affinity have been developed [85]. Based on computational and mutational studies, the binding site for the GlyT2 ligand oleoyl-D-lysine (ODLys) has been suggested to be located in a crevice between the extracellular loop 4 and trimerization domain 5 (Figure 3A) [86]. This site differs from the S1- and S2-site identified in other NTTs and suggests that NTTs can be pharmacologically modulated through a variety of different binding sites (Figure 3). ODLys has a fast brain penetration and showed activity in a rat model of neuropathic pain [87]. Importantly, ODLys displayed a unique, fast reversibility when compared with other GlyT2 inhibitors [87]. Some of these lipids described can act as partial, noncompetitive inhibitors, reaching maximal levels of inhibition that range from 50 to 90%. It should be emphasized that partial inhibition is an underexplored concept that may provide better therapeutic outcomes, as partial inhibitors would slow clearance but not stop reuptake completely [87,88].

In the case of the EAATs, the EAAT-1 selective inhibitor UCPH-101 reduces the catalytic activity of glutamate by EAAT-1 without affecting its *K_m_*. Therefore, the pharmacology of UCPH-101 is allosteric in nature, by contrast to the nonselective and competitive inhibitory profile of DL-threo-β-benzyloxyaspartic acid (TBOA) [62]. EAATs are trimeric structures [89], each with a transport domain functioning independently of the other (Figure 3B). Mutagenesis studies have located their allosteric binding site at the rim of the trimerization domain and therefore far from the orthosteric binding site [90], suggesting that the monomers might not function as completely independent entities. The observations are supported by an outward-facing EAAT-1 crystal structure in complex with UCPH-101 [62]. Despite a hypothetical benefit of UCPH-101, for instance, by blockage of exaggerated EAAT-1 mediated glutamate efflux to alleviate negative effects of anoxia or ischemia [38], its use is confined to in vitro research because of its poor blood–brain barrier permeability [91].

#### PAMs: Boosting Transporter Activity

The development of PAMs for transporters may spark great interest, specifically when it comes to transporters involved in the buffering of excitatory neurotransmitters such as glutamate or aspartate. PAMs may thus reduce excitotoxic neuronal damage [92]. They can influence membrane potential either via electrogenic transport, or through generation of an uncoupled conductance [93,94]. An example of an NTT potentiator is the venom extract of the spider Parawixia bistriata, known as Parawixin1 or PbTx1.2.3. Parawixin1 is an EAAT-2 selective potentiator. It enhances glutamate uptake in cortical synaptosomal preparations, therefore promising to protect against neuronal death in a rodent model of induced ischemia [95]. Further studies have proposed that Parawixin1 acts by accelerating a potassium-dependent return step of the transporter [96]. The Parawixin1 binding site has been localized to the EAAT2 trimerization domain [97], which has also been involved in the binding of UCPH-101, a recently developed NAM of EAAT1 (Figure 3B). This suggests that this part of the EAAT proteins could bear an allosteric site that could be targeted for the development of novel EAAT allosteric modulators [98]. Unfortunately, the exact components of the Parawixin1 extract are still unknown, but by making use of the extensive mutagenesis study carried out for Parawixin1, it was possible to tailor a series of compounds interacting with the EAAT2 trimerization domain [97]. One of these compounds, GT949 (Figure 3B), showed results comparable with Parawixin1 in increasing EAAT2 uptake [99].

In SERT, mutagenesis studies in extracellular loops 1 and 3 established residues that control transporter function [100,101]. By a virtual screen of several SERT-inhibitors, the ligand ATM7 was identified; this is able to reduce both *B_max_* and KD of [^3^H]paroxetine binding, consistent with an allosteric mechanism of action or an atypical inhibitor mode of action, binding to the orthosteric binding site in an inward-facing SERT. Moreover, cysteine accessibility analysis revealed that ATM7 stabilizes the outward-open conformation of SERT [102]. Finally, ATM7 increased 5-HT-transport and potentiates MDMA (ecstasy)-triggered reverse transport by SERT [102], thus indicating that ATM7 changes energy barriers along the path of the transport cycle.

#### Mixed NAM–PAM Behavior: The Complex Case of Zinc

Zinc (Zn^2+^) is present in many parts of the brain and modulates a number of receptors and transporters and is thus involved in regulating neurotransmission [103]. Among the group of NTTs, it has been shown that Zn^2+^ modulates the reuptake and release process of DAT, but not SERT or NET [104,105], and it also modulates glutamate and glycine transporters [106–109].

In DAT, but not SERT or NET, four residues at the extracellular side (Glu206, His193, His 375, and Asp 296) coordinate a binding site for Zn^2+^ [110–112]. The Zn^2+^ binding site is completely segregated from the substrate binding site and from the S2-site in the extracellular vestibule of NTTs (Figure 3A). Binding of Zn^2+^ to DAT elicits multiple functions: it stabilizes the DAT outward-open conformation and thereby promotes the binding of cocaine, which likely stabilizes the same conformation [42,112]. In addition, the redistribution of the conformational ensemble towards an outward-open state by Zn^2+^ binding has pronounced effects on dopamine transport [104,110], eliciting a biphasic effect with transport stimulatory properties at low concentrations and inhibitory properties at higher concentrations [19]. However, even though Zn^2+^ inhibits dopamine transport in the higher concentrations, it is inhibited to about 50–75% of the initial transport velocity [30,113] at low concentrations. Only millimolar Zn^2+^ concentrations will completely inhibit dopamine transport, probably by an indirect toxic effect. The potentiation of transport velocity by Zn^2+^ [30,113] opens up the possibility that compounds could increase dopamine clearance, and also, for example, rescue loss-of-function DAT mutants or alleviate symptoms in diseases caused by increased dopamine signaling [39]. Importantly, Zn^2+^ has been found to also bind allosterically to GlyT1, EAAT1, and EAAT4. However, in all these cases, Zn^2+^ acts as a NAM and its binding is coordinated by pairs of histidine residues located far from the orthosteric site [107–109]. Zn^2+^ could be viewed as an endogenous allosteric modulator of NTTs, as has been suggested earlier [114]. A detailed understanding of this complex mechanism of action of this important trace element may form the basis for the development of small molecules able to act as NTT potentiators.

## Concluding Remarks and Future Perspectives

Allosteric sites have been largely exploited in pharmacology and especially in the field of GPCRs and ion channels [50,115]. Targeting of such so-called ‘secondary sites’ allows tailoring of drugs around less conserved protein regions, which can potentially avoid off-target effects [50]. The rather high degree of conservation of the orthosteric binding sites in NTTs challenges exclusive targeting of specific transporter subtypes. Even if the selective serotonin reuptake inhibitors were not developed as allosteric modulators, some of them have shown moderate affinity for the S2-site. The S1- and S2-sites have been shown to be allo- sterically coupled, at least in SERT [71]. However, whether there is a physiological role for the S2-site remains unresolved and future work will be required (see Outstanding Questions). In this sense, the recent discovery of Lu AF60097 [71] will help to elucidate the role of the S2-site in SERT and its therapeutic potential. Moreover, the exact positioning of allosterically active compounds within a given NTT is still elusive, to a large extent, and needs to be assessed by a variety of different methodologies in parallel (see Outstanding Questions).

In addition, the coupling between transporter-mediated substrate uptake and efflux has still to be clarified: the fact that an abnormal basal efflux is seen in pathophysiological conditions, that different compounds can selectively target uptake, efflux or both, suggests a complex interplay between uptake and efflux that may be amenable to the development of innovative therapeutics (see Outstanding Questions). The existence of transporter inhibitors that selectively modulate only one of the two components [73] emphasizes the need to explore the substrate transport cycle in further detail [14]. In addition, abnormal efflux of neurotransmitters has been found after ischemic events [38,116], or with point mutations of NTTs associated with neuropsychiatric disorders [40,41]. Unfortunately, while the transport cycle is receiving ample attention in the case of monoamine transporters, it has not been thoroughly explored in the case of other NTTs. For instance, it is still not known which part of the transport cycle is stabilized by the EAAT-1 inhibitor UCPH-101 and which by the GlyT1 inhibitor bitopertin. Nevertheless, addressing the possibility of specifically ‘locking’ the transporter in one or more steps of its conformational cycle would be very relevant; there are several different examples where distinct conformational steps exist which could be targeted [16,18,19] (see Outstanding Questions).

To date, the only clinically approved NTT inhibitors with allosteric properties belong to the family of antidepressants (Table 1) and the clinical relevance of their allosteric modulatory effect is still not fully understood. Indeed, compared with GPCRs and ion channels, allosteric modulation of NTTs has received too little attention in past years [117], emphasizing the need to understand the function of their regulatory site(s). Moreover, many secondary sites exist in different NTTs, which may exert modulatory functions and may be targeted by exogenous or endogenous modulators: the Zn2^+^ binding site discovered in DAT, EAAT1, EAAT4, and GlyT1 serves as an example of the latter. The implementation of alternative tools for understanding how drugs can affect the transport cycle, in combination with classical pharmacological assays, may help in dissecting specific compound-related features and develop new therapeutic strategies towards allosteric modulation in a rational manner (see Outstanding Questions). Above all, an enhanced knowledge of allosteric modulation of NTTs may guide drug development processes and lead to novel therapeutic strategies, most warranted in the area of neurological and neuropsychiatric disorders [118].

## Figures and Tables

**Figure I:**
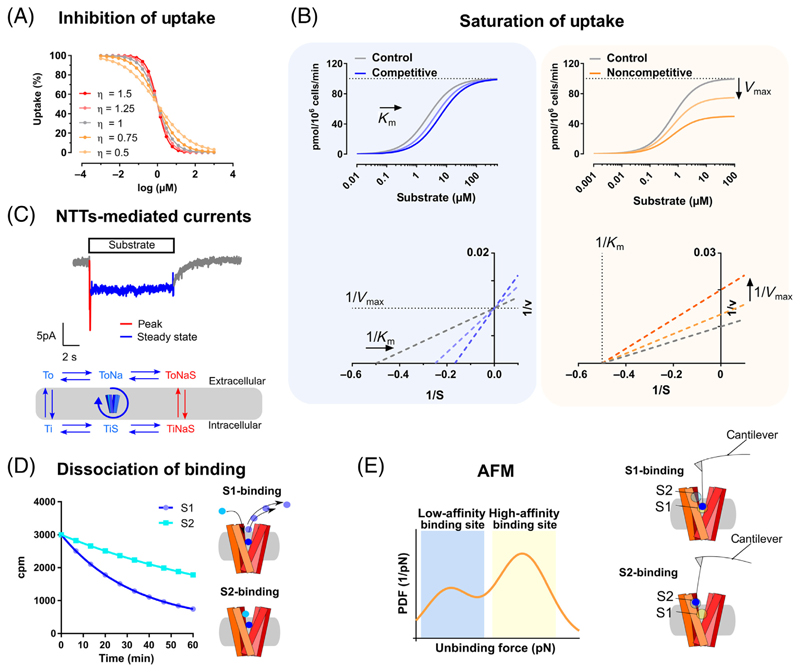
Methods to Study Allosteric Modulation of Transporters. (A) Representative graph shows depiction of Hill-Langmuir equation with change in the slope of the sigmoidal curve as a function of the cooperativity η. (B) Saturation of uptake experiments can be combined with nonlinear regression to calculate *K_m_* and *V_max_*, allowing for the identification of the specific type of allostericmodulation. Common types are competitive inhibition (left) and noncompetitive (right) allostericmodulation. In the insets, the respective Lineweaver-Burk plots are displayed. The use of nonlinear regression to identify the type of allosteric modulation has partially substituted the use of different types ofMichaelis-Menten linearization for the calculation of *K_m_* and *V_max_*. However, linearization is still widely used, because it allows a visual assessment of changes in *K_m_* and *V_max_* (B, bottom panels). In addition to the Lineweaver-Burk plot [133], the Hofstee [134] or the Eadie-Hofstee method [135] are also commonly used. (C) Representative trace of transporter-mediated current measured by electrophysiology and a simplified version of the transport cycle is shown. Note that different components of the current correspond to different partial reactions (blue versus red in the scheme). (D) Representative graph showing the reduction in the dissociation half-life of a radioactive tracer (S1, blue) mediated by the binding of an allosteric modulator (S2, cyan). (E) Schematic representation of the interaction between the binding sites of the transporter and the motion-sensitive cantilever (highlighted) with the attached ligand [bottom: S1-site (= orthosteric site), top: S2-site (= allosteric site)]. The probability of unbinding the ligand from these sites can be plotted against the force applied to obtain a unimodal or multimodal distribution. This depends on whether one or more sites are involved in the binding of the ligand. Abbreviations: NTT, neurotransmitter transporters; PDF, probability density function.

**Table 1:** List of the Currently Available Allosteric Modulators of NTTs with Their Alternative Names, Promising Preclinical Features, and Examples of Clinical Testing^a^

Structure	Compound	Target	Uptake	Efflux	Activity	Stage	Refs
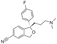	S- Citalopram or escitalopram	SERT	↓	↓	NAM	Approved	[64,66,119]
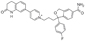	Lu AF60097	SERT	↓	n.d.	NAM	Preclinical	[71]
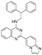	SRI-29574	DAT (also NET/SERT)	↓	–	NAM	Preclinical	[60]
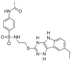	KM822	DAT	↓	n.d.	NAM	Preclinical	[75]
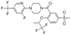	Bitopertin or RG1678; RO4917838	GlyT1	↓	n.d.	NAM	Phase III	[76,79,82]
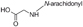	*N*-arachidonyl-glycine (NAGly)	GlyT2	↓	n.d.	NAM	Preclinical	[86,87]
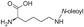	*N*-oleoyl-D-lysine (ODLys)	GlyT2	↓	n.d.	NAM	Preclinical	[86,87]
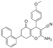	UCPH-101	EAAT1	↓	n.d.	NAM	Preclinical	[62,90]
n.d.	Parawixin1 (PbTx1.2.3)	EAAT2	↑	–	PAM	Preclinical	[96]
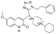	GT949	EAAT2	↑	n.d.	PAM	Preclinical	[99]
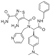	ATM7	SERT	↑	↑	PAM	Preclinical	[102]

a Abbreviations: DAT, dopamine transporter; EAAT, excitatory amino acid transporter; GlyT, glycinetransporter; NAM, negative allosteric modulator; n.d., not determined; NET, norepinephrine transporter; PAM, positive allosteric modulator; SERT, serotonin transporter, ↓, reduced; ↑, increased; –, no change.

## Box 1 Methods for Studying Allosteric Modulation of Transporters

### Hill-Langmuir Equation

The Hill-Langmuir equation describes the occupancy of a receptor by its ligand.

$$\theta =\frac{{\left[\text{L}\right]}^{\eta }}{K_{\text{d}}+{\left[L\right]}^{\eta }}=\frac{1}{1+{\left(\frac{K_{A}}{\left[L\right]}\right)}^{\eta }}$$

where θ is the fraction of the receptor–ligand complex, [L] is the concentration of unbound ligand, Kd is the dissociation constant, Ka is the ligand concentration producing half occupation, and η is the Hill coefficient assuming any positive value [53,131]; η describes the degree of cooperativity between multiple binding sites for the same ligand. With η = 1 there is no cooperativity, with η N 1 the cooperativity is positive, and with η b 1 it is negative [53](Figure IA).

### Uptake (Saturation) Experiments

Classical uptake-saturation experiments are conducted in the presence of different concentrations of a putative allosteric ligand. The Michaelis-Menten parameters (*K_m_* and *V_max_*) are derived from the dose response curves using a linearized form of the Lineweaver-Burk plot or the Eadie-Hofstee diagram. Direct nonlinear regression analysis of the dose response data allows for alleviating the limitations of the linearized equations. Extrapolation of the Lineweaver-Burk plot to the abscissa for two measurements in the presence or absence of a second ligand allows for a simple detection of competitive or noncompetitive interaction (Figure IB).

Alternatively, coupling between substrate and ions during the transport process generates transporter-mediated currents (measured electrophysiologically), which can be used as a surrogate of NTT activity (Figure IC). The whole-cell patch clamp approach provides access to the internal side of the cell. By changing the ionic compositions inside and outside of the cell, it is possible to isolate different components of the current (i.e., the steady-state, representing an average of all the transport partial reactions, and the peak, representing the outward-open, ToSNa, to inward-open transition, TiSNa) and to study partial reactions of the transport cycle [14,23] (Figure IC).

### Dissociation Experiments

Allosteric ligands can change the dissociation of a prebound inhibitor. In brief, membrane preparations containing the protein of interest are incubated with a high affinity [^3^H]inhibitor. After the equilibrium is reached, the ligand of interest is added to the preparation and the reaction is stopped at different time-points. If the ligand binds to an allosteric site that increases the affinity in the central binding site, the dissociation rate is reduced [66] (Figure ID).

### Atomic Force Microscopy (AFM)

AFM can be used to study interactions of ligands and proteins at a single molecule level in native tissues. In brief, the tip of a highly sensitive cantilever is functionalized with the putative allosteric ligand and incubated with the cells expressing the desired transporter. Once at equilibrium, the AFM tip is lifted up and the bending of the cantilever allows the quantification of distribution of forces. A binomial distribution is indicative of a secondary binding site [69,132] (Figure IE).

## Box 2 Overview of Different Allostery Models

At the start of the 20th century, changes in pH or in the concentration of CO_2_ were found to modulate oxygen binding to hemoglobin [120]. Later, work on the feedback mechanism of l-threonine deaminase revealed the existence ofadifferent and nonoverlapping binding site acting ‘at-a-distance’ on the central binding site [47]. Subsequently, the process was termed ‘allosteric’ binding [48]. These observations initiated the conceptualization of the conformation-selection model, also known as the ‘Monod-Wyman-Changeux’ (MWC) model, which assumes that ligands prefer one of the two states that the protein adopts at equilibrium. These two states are commonly termed the ‘relaxed’ (R) and ‘tensed’ (T)state. Upon ligand-binding, one conformation is selected. As the transitions between the R and T state are cooperative and include all protomers, ligand binding transduces a signal to all protomers through few discrete conformations [54]. As an alternative to the MWC-model, Koshland, Nemethy, and Filmer proposed the ‘induced-fit’ model (or KNF model). This model predicts a structural change only in response to binding of one or more ligands, which is symmetrically communicated to all protomers [121]. Both the conformational-selection and the induced-fit models rely on a series of discrete conformational rearrangements, without considering changes in the energy landscape involved in allosteric modulation. As such, they were limited in explaining a number of effects. (i) Allosteric effects can occur without obvious structural changes [122,123]. (ii)Mutations of surface exposed residues can modulate thefunction ofthe active site, whilenotshowing stable interactions with other residues [124,125]. (iii) Allosteric signal transmission between the allosteric and the orthosteric site can also occur in the absence of an obvious connection between the two sites [126,127]. (iv) Binding of ligands to the orthosteric or allosteric site may not be correlated with protein stability changes [128,129]. (v) Negative **cooperativity** may not be described by the induced-fit or conformational-selection model [128].

To overcome the limitation of excluding energetic considerations, the ‘ensemble allostery model’ and the ‘allosteric Ising model’ (AIM) have been proposed [55,130]. AIM, in particular, bridges thermodynamic and structural aspects using a statistical ensemble approach.

## Box 3 Modulation of Efflux: Positive and Negative Modulatory Effects

The PAM ATM7 has been shown to increase MDMA-triggered, SERT-mediated [^3^H]5-HT release [102]. SoRI-9804 and SoRI-20040,instead,can work as NAM of both uptake and efflux[73].However,Parawixin1 and SRI-29574 allosterically inhibit EAAT2- and DAT-uptake, respectively, without affecting efflux [60,96]. The complex interplay between uptake and efflux has been observed also in the case of the SERT-selective, para-trifluoromethyl-methcathinone (p-CF3-MCAT). A point mutation in the S2-site of SERT could modulate the p-CF3-MCAT-mediated **partial release** of [^3^H]5-HT but not the **full release** mediated by para-chloro-amphetamine, suggesting that the S2-site of SERT may work as a regulatory site for substrate efflux [29].

In addition, the endogenous trace element Zn^2+^ has been shown to enhance DAT-mediated, amphetamine-induced re-lease[104,113].It has been suggested that Zn^2+^may work as an endogenous modulator of dopamine neurons with re-spect to their firing frequency by enhancing DAT-mediated release of dopamine [114].

Drug-induced efflux has been studied mainly at monoamine transporters,as discussed above. In the case of a large pro-portion of all osteric NTT modulators,the effect on efflux was not determined.This may have to do with:(i)the fact that efflux is still an understudied phenomenon for some NTTs and/or ligands; and (ii) ‘amphetamine-type’ releasers are not available for a large number of transporters.In the case of EAAT1,reverse transport has mainly been described under pathophys-iological conditions [38,116] and therefore the development of compounds leading to an increase in extracellular glutamate seemed pointless, if not disadvantageous, with respect to excitotoxicity. Consequently, for several, if not most, compounds, only the uptake has been characterized (see main text for discussion).

## Highlights

Current research is showing an increasing heterogeneity in the binding sites available in NTTs. These binding sites are often located distant from the central binding sites, suggesting a higher level of complexity in the regulation of NTT function.

Allosteric sites increase specificity, reducing off-target effects.

Small molecules can be developed to target NTT allosteric sites, with the possibility to either increase or decrease their functions.

NTT allosteric modulators are in advanced clinical trials, emphasizing their potential for the treatment of neuropsychiatric disorders.

## Glossary

### Abbreviations

Cooperativity: the effect a ligand has on the binding of a second ligand.
Full release: pharmacological agents like d-amphetamine, methamphetamine, *para*-chloro-amphetamine, or MDMA can elicit a pronounced efflux of neurotransmitters, also known as full release.
Partial release: some pharmacological agents can mediate a reduced maximal efflux of neurotransmitter (partial release) when compared with a full-releaser. Partial releasers are included in the nonclassical pharmacology of NTTs.
Use-dependency: the ability of a drug to bind its target only when in a specific state, (e.g., only when it is in its active form or ‘in use’).

## Outstanding Questions

Does the allosteric modulation of NTTs have a physiological role?

How can we ascertain allosteric binding sites in NTTs and pinpoint the exact interaction partners?

Is it possible to implement the transport cycle into the drug-development process?

Is it possible to develop compounds that specifically stabilize specific conformations of the transporter? And how are the different conformations of NTTs transducing the signal into the cell?

Is it possible to allosterically modulate transporter-mediated efflux? How could this be used pharmacologically/clinically?

## Resources

i
https://clinicaltrials.gov/ct2/show/NCT01674361

ii
https://clinicaltrials.gov/ct2/show/NCT03271541
